# Blackberry Juice Fermented with Two Consortia of Lactic Acid Bacteria and Isolated Whey: Physicochemical and Antioxidant Properties during Storage

**DOI:** 10.3390/ijms25168882

**Published:** 2024-08-15

**Authors:** Liliana Lugo-Zarate, Luis Delgado-Olivares, Nelly del Socorro Cruz-Cansino, Luis Guillermo González-Olivares, Nayeli Shantal Castrejón-Jiménez, Diego Estrada-Luna, Angélica Saraí Jiménez-Osorio

**Affiliations:** 1 Área Académica de Enfermería, Instituto de Ciencias de la Salud, Universidad Autónoma del Estado de Hidalgo, Circuito Actopan Tilcuautla s/n. Ex Hacienda La Concepción, San Agustín Tlaxiaca 42160, Hidalgo, Mexico; lu279379@uaeh.edu.mx (L.L.-Z.); destrada_luna@uaeh.edu.mx (D.E.-L.); 2 Área Académica de Nutrición, Instituto de Ciencias de la Salud, Universidad Autónoma del Estado de Hidalgo, Circuito Actopan Tilcuautla s/n. Ex Hacienda La Concepción, San Agustín Tlaxiaca 42160, Hidalgo, Mexico; ldelgado@uaeh.edu.mx (L.D.-O.); ncruz@uaeh.edu.mx (N.d.S.C.-C.); 3 Área Académica de Química, Instituto de Ciencias Básicas e Ingeniería, Universidad Autónoma del Estado de Hidalgo, Mineral de la Reforma 42184, Hidalgo, Mexico; lgonzales@uaeh.edu.mx; 4 Área Académica de Medicina Veterinaria y Zootecnia, Instituto de Ciencias Agropecuarias, Universidad Autónoma del Estado de Hidalgo, Av. Universidad km 1 Ex Hacienda de Aquetzalpa A.P. 32, Tulancingo 43600, Hidalgo, Mexico; nayeli_castrejon@uaeh.edu.mx

**Keywords:** lactic acid bacteria, fermentation, blackberry juice, whey, antioxidants

## Abstract

Fermenting fruit juices with lactic acid bacteria (LAB) is a sustainable method to enhance fruit harvests and extend shelf life. This study focused on blackberries, rich in antioxidants with proven health benefits. In this research, we examined the effects of fermentation (48 h at 37 °C) at 28 days on whey-supplemented (WH, 1:1) blackberry juice (BJ) inoculated with two LAB mixtures. Consortium 1 (BJWH/C1) included *Levilactobacillus brevis*, *Lactiplantibacillus plantarum*, and *Pediococcus acidilactici*, while consortium 2 (BJWH/C2) comprised *Lacticaseibacillus casei* and *Lacticaseibacillus rhamnosus*. All of the strains were previously isolated from aguamiel, pulque, and fermented milk. Throughout fermentation and storage, several parameters were evaluated, including pH, lactic acid production, viscosity, stability, reducing sugars, color, total phenolic content, anthocyanins, and antioxidant capacity. Both consortia showed a significant increase in LAB count (29–38%) after 16 h. Sample BJWH/C2 demonstrated the best kinetic characteristics, with high regression coefficients (R^2^ = 0.97), indicating a strong relationship between lactic acid, pH, and fermentation/storage time. Despite some fluctuations during storage, the minimum LAB count remained at 9.8 log CFU/mL, and lactic acid content increased by 95%, with good storage stability. Notably, sample BJWH/C2 increased the total phenolic content during storage. These findings suggest that adding whey enhances biomass and preserves physicochemical properties during storage.

## 1. Introduction

In recent decades, there has been a growing focus on researching and developing alternative foods and production processes that are viable for human consumption and beneficial to health [1,2]. Fermentation, an ancient process involving the biotransformation of fermentable substrates, such as carbohydrates primarily or proteins, through interactions with bacteria or fungi, has enhanced food’s sensory, storage, and nutritional qualities in both industrial and home settings. Additionally, fermentation has proven highly effective in developing functional foods that contain a wide range of metabolites, including organic acids, free amino acids, short-chain fatty acids, bioactive peptides [2,3], volatile compounds, and other substances, that can alter the intestinal microbiota [4]. As a result, many foods with probiotic, antimicrobial, and antioxidant properties have been developed [5,6].

In fruits, natural and spontaneous fermentation is driven by lactic acid bacteria (LAB), such as *Lactobacillus* spp., *Leuconostoc* spp., *Pediococcus* spp., *Weisella* spp., and other autochthonous strains, due to the presence of carbohydrates [7]. On an industrial scale, the use of LAB, like *Lactiplantibacillus plantarum*, *Lacticaseibacillus rhamnosus*, *Lacticaseibacillus casei*, *Lactobacillus gasseri*, and *Lactobacillus acidophilus*, enhances the antioxidative and antibacterial properties of fermented products [8,9,10,11,12] and regulates the levels of secondary metabolites and final products. Functional foods, particularly red fruits, are also explored in fermentation due to their low sugar content and high polyphenolic compound concentration [13]. Fermentation with red fruits has shown significant growth of LAB, including *L. plantarum*, *Pediococcus acidilactici*, *Pediococcus pentosaceus*, and *Leuconostoc mesenteroides* subsp. *mesenteroides*, alongside lactic acid production. However, certain fruits, such as blackberry, possess notable antioxidant, antimicrobial, anti-inflammatory, and antidiabetic properties due to their organic acid content and low pH levels, which can inhibit LAB growth and lactic acid production [14].

On the other hand, whey, a byproduct of milk derivatives, is often considered an organic residue in the industry. However, it has been shown to enhance nutritional quality and promote sustainable food production [15]. This is primarily due to its high protein content, including α-lactalbumin, β-lactoglobulin, immunoglobulins, and lactoferrins [16]. Whey fermentation produces bioactive peptides, exopolysaccharides, organic acids, and antioxidants [17]. Furthermore, whey has been found to enhance antimicrobial activities when combined with certain fruits [18]. Research has also indicated that whey protein can improve glucose levels [19], which may be linked to the stability of phenols and anthocyanins through molecular interactions between antioxidants and whey proteins [20]. Consequently, incorporating whey protein into a matrix could influence LAB substrates and the stability of antioxidant and antibacterial properties during storage.

In fermented foods like yogurt, a decline in LAB concentration during storage at 4 °C for up to 28 days has been observed [21]. However, in fermented juices, such as blackberry juice fermented with *Lactobacillus*, the phenolic content and antioxidant properties have shown improvement under similar conditions [22]. Thus, this study aimed to assess the LAB growth, physicochemical characteristics, and antioxidant capacity of blackberry (*Rubus fruticosus*) juice fermented with whey isolated from milk and inoculated with two different LAB consortia.

## 2. Results

### 2.1. Fermentation for 24 and 48 Hours

**Figure 1:**
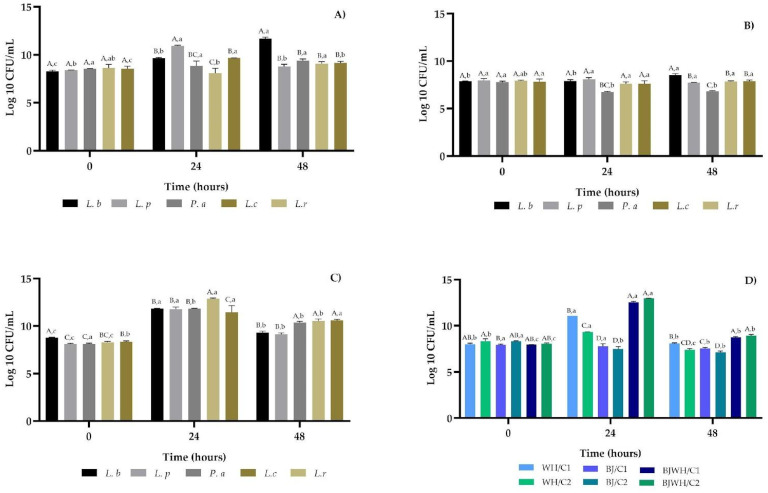
Growth of LAB during fermentation at 0, 24 and 48 h. Individual strains: *Levilactobacillus brevis* (L. b), *Lactiplantibacillus plantarum* (L. p), *Pediococcus acidilactici* (P. a), *Lacticaseibacillus rhamnosus* (L. r), and *Lacticaseibacillus casei* (L. c) were inoculated and fermented in WH (whey protein) (**A**), BJ (blackberry juice) (**B**), and BJWH (blackberry juice + whey protein) (**C**). WH/C1 (whey protein inoculated with consortium 1: L. b, L. p and P. a), WH/C2 (whey protein inoculated with consortium 2: L. r and L. c), BJ/C1 (blackberry juice with consortium 1), BJ/C2 (blackberry juice with consortium 2), BJWH/C1 and BJWH/C2 (blackberry juice + whey protein with consortium 1 and 2, respectively) (**D**). Values represent three experiments’ mean ± standard deviation (n = 3). Different capital letters indicate a significant difference (*p* < 0.05) between the different strains or consortium for each fermentation time. Different lowercase letters indicate a significant difference (*p* < 0.05) of each strain or consortium between their different fermentation times.

The lactic acid fermentation experiment involved the controlled inoculation of five individual LAB strains, previously isolated from aguamiel (*L. plantarum* and *Pediococcus acidilactici*), pulque (*Levilactobacillus brevis*), and commercial fermented milk *(Lacticaseibacillus casei* and *Lacticaseibacillus rhamnosus*). These strains have demonstrated inhibitory effects against *Escherichia coli*, *Staphylococcus aureus*, and *Helicobacter pylori* [23]. Each strain was inoculated at a concentration of 9 log CFU/mL into three different fermentation systems: milk whey (WH), blackberry juice (BJ), and a 1:1 mixture of WH and BJ over a 48-h period at 37 °C (Figure 1A–C).

The results showed that *L. brevis* exhibited the highest growth in the WH system, reaching 11.68 ± 0.15 Log 10 CFU/mL at 48 h, followed by *L. plantarum*, which reached 10.90 ± 0.09 Log 10 CFU/mL at 24 h (Figure 1A). In the BJ system, the strains maintained their initial LAB counts throughout the fermentation process (Figure 1B). However, all strains showed significantly increased growth in the mixture of BJ and WH (1:1), averaging 11.94 Log 10 CFU/mL at 24 h (Figure 1C).

Two fermentation systems were developed from the BJWH mixture to compare the effects of LAB strains isolated from pulque and aguamiel (*L. brevis*, *L. plantarum*, and *P. acidilactici*) with those isolated from commercial products (*L. rhamnosus* and *L. casei*). This led to the creation of two consortia: consortium 1 (C1) consisted of *L. brevis*, *L. plantarum*, and *P. acidilactici*, while consortium 2 (C2) included *L. casei* and *L. brevis* (C2). C1 and C2 were inoculated into the three fermentation systems (WH, BJ, and BJWH). The highest growth was observed in both consortia BJWH/C1 and BJWH/C2 at 24 h, with 12.54 ± 0.10 Log 10 CFU/mL and 12.94 ± 0.04 Log 10 CFU/mL, respectively, followed by the WH/C1 system (Figure 1D).

### 2.2. Physicochemical Properties of Fermented Samples

After fermentation, pH, lactic acid content, total soluble solids (°Brix), and reducing sugars were measured to assess bacterial growth and the final products of LAB metabolism. The results of these physicochemical properties during the fermentation process (at 0, 24, and 48 h) are presented in Table 1.

Initially, the BJWH system had the lowest pH values, ranging from 5.4 to 5.6. After 24 h, the WH/C2, BJ/C1, BJWH/C1, and BJWH/C2 systems exhibited significantly lower pH values. By 48 h, the pH values remained lower in the BJ/C1, BJWH/C1, and BJWH/C2 systems. The lactic acid percentage increased notably in the BJ/C1 system, followed by BJWH/C1 and BJWH/C2, while the WH/C1 and WH/C2 systems showed no significant changes at 24 and 48 h. This increase in lactic acid was associated with a decrease in total soluble solids by one unit at 24 and 48 h for the BJWH/C1 and BJWH/C2 systems. Additionally, reducing sugars were initially lower in the BJWH systems and decreased most significantly in the BJWH/C1 system by 48 h (Table 1).

### 2.3. Physicochemical Properties during Storage

**Figure 2:**
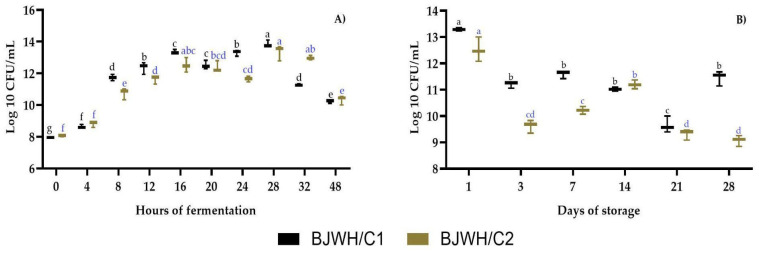
LAB growth during (**A**) fermentation of 48 h and (**B**) storage days after 16 h of fermentation of BJWH/C1 and BJWH/C2 (blackberry juice + whey protein with consortium 1 and 2, respectively). The sample contains C1: *Levilactobacillus brevis*, *Lactiplantibacillus plantarum*, and *Pediococcus acidilactici* and C2: *Lacticaseibacillus casei* and *Lacticaseibacillus rhamnosus*. Values in each point represent three experiments’ mean ± standard deviation (n = 3). Different black (BJWH/C1) and blue (BJWH/C2) lowercase letters indicate a significant difference (*p* < 0.05) in different fermentation and storage times.

The endpoint of fermentation was assessed by measuring CFU at intervals 0, 4, 8, 16, 20, 24, 28, 32, and 48 h of incubation at 37 °C for BJWH/C1 and BJWH/C2. Figure 2A shows LAB growth, with BJWH/C1 exhibiting a notable increase from 16 h (13.34 ± 0.14 Log 10 CFU/mL) to 28 h (13.85 ± 0.21 Log 10 CFU/mL). However, a decrease in the LAB count was observed at 20 h for both BJWH/C1 and BJWH/C2, followed by variable behavior in subsequent hours, finally resulting in a lower average count (10.27 Log 10 CFU/mL) at 48 h. Consequently, storage analysis was initiated after 16 h of fermentation of the BJWH/C1 and BJWH/C2 samples.

Fermented samples were stored at 4 °C, and LAB counts were assessed on storage days 3, 7, 14, 21, and 28. While growth patterns varied among samples, BJWH/C1 consistently exhibited higher LAB counts, averaging 11.29 Log 10 CFU/mL on days 3, 7, 14, and 28. Notably, BJWH/C2 showed an increase in LAB count on day 14, followed by a significant decline by day 28 (see Figure 2B).

Kinetic parameters were calculated to determine significant differences between fermentations (see Table 2). BJWH/C2 exhibited more favorable parameters, including a longer generation time than BJWH/C1 (4.20 ± 0.60). However, the fermentation rate (µ) was faster in BJWH/C1, while BJWH/C2 demonstrated a better affinity, as indicated by its constant (k). These observations suggest a higher cellular density in BJWH/C2, particularly in systems containing *L. casei* and *L. rhamnosus*.

**Figure 3:**
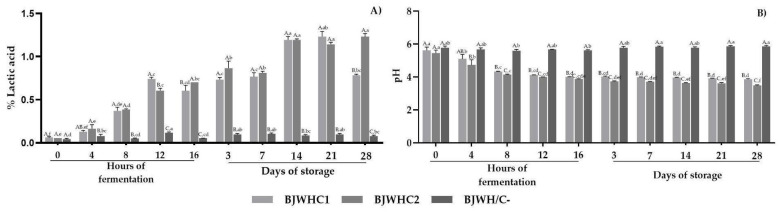
Lactic acid (**A**) and pH (**B**) values across the time of fermentation (16 h) and the time of storage (28 days) in Blackberry juice + whey protein inoculated with consortium 1 of *Levilactobacillus brevis*, *Lactiplantibacillus plantarum*, and *Pediococcus acidilactici* (BJWH/C1), consortium 2 of *Lacticaseibacillus casei* and *Lacticaseibacillus rhamnosus* (BJWH/C2) and the negative control without LAB inoculum (BJWH/C-). Values represent three experiments, mean ± standard deviation (n = 3). Different capital letters indicate significant differences (*p* < 0.05) between the various strains or consortiums for each fermentation and storage time. Different lowercase letters indicate a significant difference (*p* < 0.05) of each strain or consortium between their different fermentation and storage times.

The percentages of lactic acid and pH values were measured from the start of fermentation to the end of the storage (day 28, Figure 3). Lactic acid production rose after 4 h of fermentation for BJWH/C1 (0.12%) and BJWH/C2 (0.16%). The highest levels of lactic acid were observed in the BJWH/C2 group during the final 14 days of storage (Figure 3A). Additionally, while the initial pH averaged 5.6 throughout fermentation, it decreased significantly in the BJWH/C2 system during fermentation and storage. BJWH/C1 showed a substantial reduction in pH within the first eight hours of fermentation but remained relatively stable from 12 h onwards through the storage period (Figure 3B).

**Figure 4:**
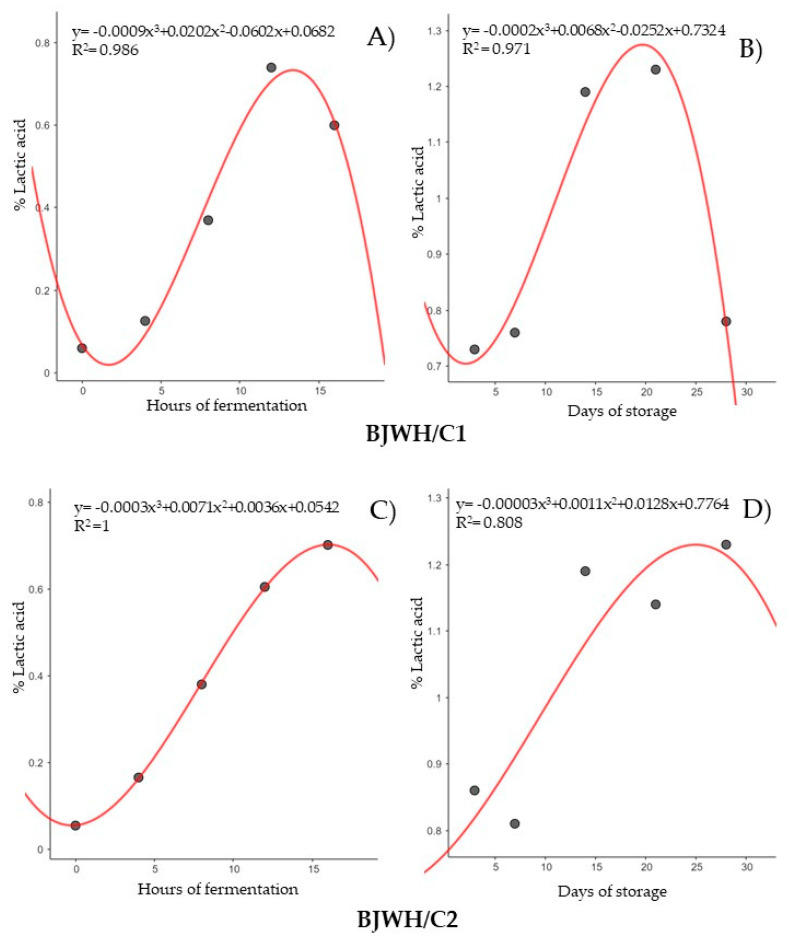
Non-linear regression analysis lactic acid values across the time of fermentation (16 h) and the time of storage (28 days) in blackberry juice + whey protein inoculated with consortium 1 of *Levilactobacillus brevis*, *Lactiplantibacillus plantarum*, and *Pediococcus acidilactici* (BJWH/C1) (**A**,**B**), consortium 2 of *Lacticaseibacillus casei* and *Lacticaseibacillus rhamnosus* (BJWH/C2) (**C**,**D**).

**Figure 5:**
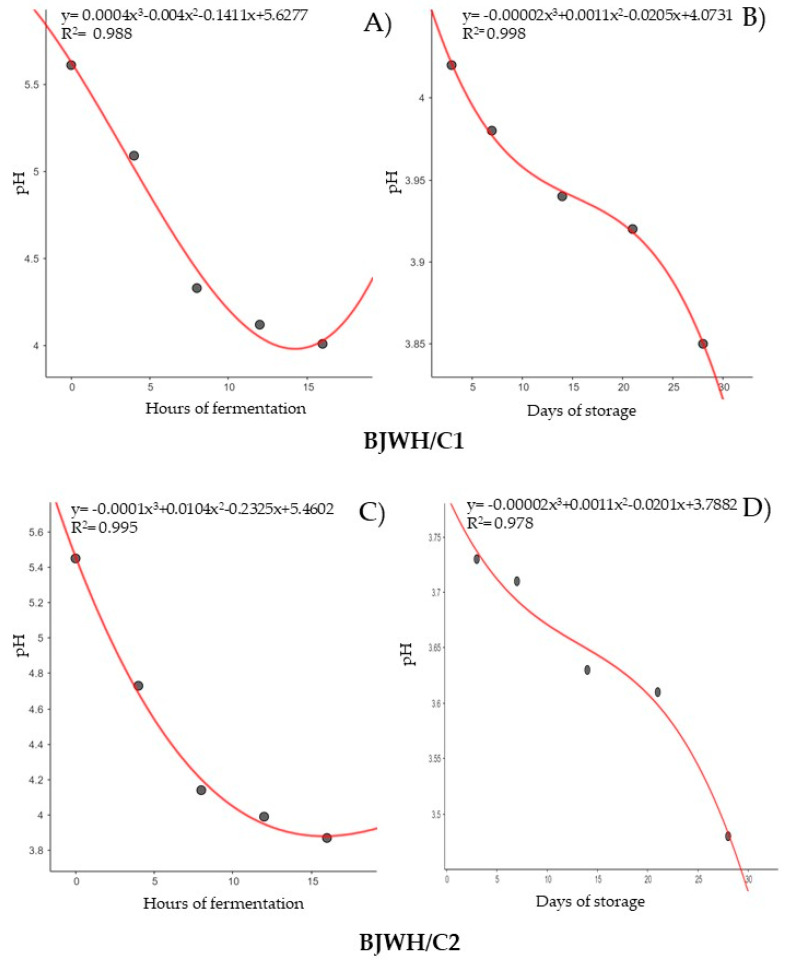
Non-linear regression analysis pH values across the time of fermentation (16 h) and the time of storage (28 days) in blackberry juice + whey protein inoculated with consortium 1 of *Levilactobacillus brevis*, *Lactiplantibacillus plantarum*, and *Pediococcus acidilactici* (BJWH/C1) (**A**,**B**), consortium 2 of *Lacticaseibacillus casei* and *Lacticaseibacillus rhamnosus* (BJWH/C2) (**C**,**D**).

Regression analysis was used to determine the correlation coefficients for lactic acid (Figure 4) and pH (Figure 5) with fermentation and storage time. Data for each factor were modeled with a third-degree polynomial curve. Lactic acid levels increased continuously up to 12 h of fermentation for BJWH/C1 (Figure 4A) and up to 16 h for BJWH/C2 (Figure 4C), with similar trends extending up to 21 days of storage for BJWH/C1 (Figure 4B). Most curves exhibited an R^2^ value greater than 0.97, except for lactic acid production in BJWH/C2 during storage (Figure 4D), which had an R^2^ of 0.80. pH levels decreased with extended fermentation or storage time, reflecting their relationship with lactic acid production (Figure 5). This analysis highlights a strong connection between lactic acid and pH, significantly influenced by time.

The total soluble solids were one °Brix lower in the LAB-inoculated systems (BJWH/C1 and BJWH/C2) than controls after the 8 h of fermentation and remained at 5 °Brix throughout storage. Reducing sugars decreased in BJWH/C1 and BJWH/C2 during fermentation and storage, with a more pronounced reduction in BJWH/C2. Overall, reducing sugars levels were significantly lower in BJWH/C1 and BJWH/C2 throughout fermentation and storage compared to the negative control, BJWH/C- (Table 3).

The acceptability of beverages is influenced by their physical stability, viscosity, and color. Table 4 shows the stability (nonprecipitated solids) and viscosity (centipoise) values at the start and end of fermentation (16 h) and during storage. At the end of fermentation and throughout, BJWH inoculated with LAB exhibited greater stability than the negative control, with a mean difference of 35% compared to BJWH/C-. The stability remained consistent for both LAB-inoculated consortia during storage. Regarding viscosity, LAB-inoculated systems (BJWH/C1 and BJWH/C2) significantly decreased in cP values after fermentation, maintaining lower viscosity levels than the negative control throughout storage. There was no significant difference in viscosity between BJWH/C1 and BJWH/C2 (Table 4).

Regarding the colorimetric characteristics, the L parameter (luminosity) varied among samples with the LAB inoculum but remained consistent after fermentation and throughout storage (Table 5). The predominant color was red, indicated by increasing a* values post-fermentation, with higher values observed in BJWH/C2 samples during storage. The b* parameter, initially negative in LAB-inoculated samples (BJWH/C1 and BJWH/C2), indicating a blue hue, shifted to positive values in BJWH/C- during storage, reflecting a change to more yellow tones.

Chroma measures color saturation, with values ranging from 0 to 100, reflecting the intensity of the sample’s color. The results show that BJWH/C2 and BJWH/C1 are brighter than BJWH/C-. Additionally, negative hue values in BJWH/C1 and BJWH/C2 indicate a pinkish tone. A more detailed graphical representation of these color results is provided in Table 5.

**Figure 6:**
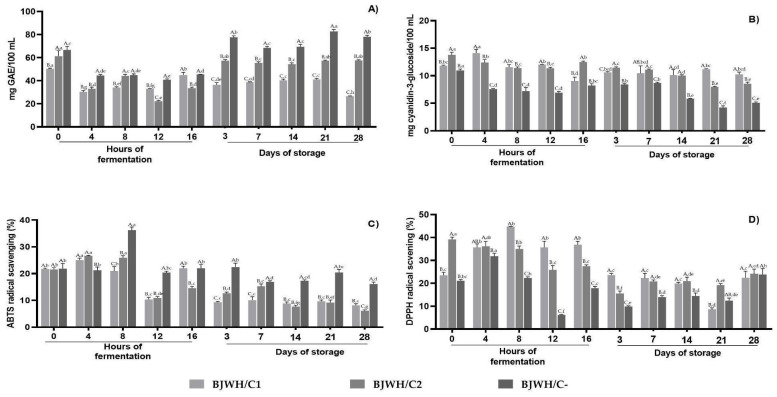
Content of total phenolic compounds (**A**), anthocyanins (**B**), ABTS scavenging (**C**), and DPPH scavenging (**D**). Values across the time of fermentation (16 h) and the time of storage (28 days) in Blackberry juice + whey inoculated with consortium 1 of *Levilactobacillus brevis*, *Lactiplantibacillus plantarum*, and *Pediococcus acidilactici* (BJWH/C1), consortium 2 of *Lacticaseibacillus casei* and *Lacticaseibacillus rhamnosus* (BJWH/C2) and the negative control without LAB inoculum (BJWH/C-). Values represent three experiments with mean ± standard deviation (n = 3). Different capital letters indicate significant differences (*p* < 0.05) between the different strains or consortiums for each fermentation and storage time. Different lowercase letters indicate a significant difference (*p* < 0.05) of each strain or consortium between their different fermentation and storage times.

Finally, the levels of phenolic compounds, anthocyanins, and the scavenging activity of 2,2-Azino-bis-3-ethylbenzothiazoline-6-sulfonic acid (ABTS) and 2,2-diphenyl-1-picrylhydrazyl (DPPH) were evaluated during fermentation and storage (Figure 6). The total phenolic content significantly decreased in all samples after 4 h of fermentation, averaging 30 mg GAE/100 mL (*p* < 0.05). This decline continued in BJWH/C1 through the end of storage, while BJWH/C2 showed a 19% increase after 3 days of storage, with higher levels observed in the control (BJWH/C-, Figure 6A). In contrast, the anthocyanin content was notably higher in BJWH/C1 after 4, 8, and 12 h of fermentation but showed an opposite trend at 16 h and 3 days. During storage, LAS-inoculated systems had a higher anthocyanin content compared to BJWH/C-, with BWH/C1 maintaining greater stability than BJWH/C2 by the end of storage (Figure 6B).

For ABTS, which measures free radical scavenging activity, the BJWH/C1 fermentation system maintained its antioxidant activity at 0 h. However, during storage, the antioxidant capacity decreased by 57.7% in both consortia, with the non-inoculated sample exhibiting a significantly higher scavenging capacity (*p* < 0.05, Figure 6C). Despite this, DPPH scavenging activity was significantly higher at 8, 12, and 16 h of fermentation in BJWH/C1, averaging 38% activity. After 3 days of storage, the scavenging activity decreased by 36% in BJWH/C1, 43% in BJWH/C2, and 44.6% in BJWH/C- (Figure 6D).

## 3. Discussion

This study explored the effects of adding isolated whey protein to blackberry juice, using two consortia to optimize biomass growth at 24 and 48 h of fermentation. Additionally, we aimed to maintain stable levels of phytochemicals and antioxidants throughout storage. The growth of strains like *L. brevis* and *L. plantarum* was significantly higher at 24 h in the whey protein system. However, the blackberry juice (BJ) system was less favorable for all LAB strains. This outcome is similar to findings from fermenting elderberry juice with *L. plantarum*, *L. rhamnosus*, and *L. casei*. The authors noted that *L. casei* strains struggled to grow in the acidic environment, likely due to their origin in cheese, making them less adapted to acidic juices [24,25,26].

In the case of the limited growth observed in the BJ system, it could be attributed to the inherent chemical and physical properties of the fruit, such as its acidic environment, buffering capacity, high concentrations of indigestible nutrients (like fiber and fructooligosaccharides), and the presence of anti-nutritional compounds (such as phenols and tannins). Additionally, these factors may interact with the amino acids and proteins in whey [27].

Therefore, a protein source has been proposed to enhance biomass production and create a supportive environment for lactic acid bacteria (LAB). However, few studies have explored the impact of adding whey protein for fermentation systems to boost biomass in fruit beverages. Our research found that fermenting blackberry juice alone did not increase LAB counts. However, adding whey protein to blackberry juice significantly raised the LAB counts after 24 h (Figure 1C). Additionally, two different consortia were used, one consisting of *L. brevis*, *L. plantarum*, and *P. acidilactici* and the other of *L. casei* and *L. rhamnosus*, which proved to be more effective in promoting growth in the BJWH fermentation system, resulting in significantly higher LAB counts than other matrices (BJ and WH).

In addition, a similar pattern was observed in whey fermentation using *L. rhamnosus* and *S. thermophilus*, both in monoculture and co-culture. The authors found co-culturing these strains was more effective for biomass production [28]. The bacterial growth observed in BJWH/C1 and BJWH/C2 could be attributed to adding whey protein as a supplement, which enhances biomass production during fermentation and provides a valuable source for functional metabolites [3].

This study showed that combining blackberry juice with whey protein resulted in a more substantial increase in biomass than using the juice alone, where LAB counts decreased after 24 h. The synergy between these two food matrices (blackberries, which provide sucrose, glucose, and fructose as energy sources for LAB [29], and whey, which supplies proteins, lactose, fat, and mineral salts [30]) promotes enhanced biomass production. Phenolic compounds, especially ferulic and phenolic esters, play a crucial role in the growth and viability of probiotics in fruit-based food matrices [7]. Their presence has been shown to positively impact them, as they are susceptible to hydrolysis by *L. plantarum* esterases, including feruloyl esterase, tannase, and gallate decarboxylase [31]. Addotionally, blackthorn extract (up to 0.62 mg) has been found to exhibit prebiotic properties that support the growth of LAB strains, such as *L. plantarum*, *L. reuteri*, *L. rhamnosus*, and *S. thermophilus* [32].

Conversely, synergism within bacterial consortia establishes the essential conditions for strain growth. Compared to individual samples, the notable bacterial growth in BJWH/C1 and BJWH/C2 can be attributed to the LAB protocooperation system. In these systems, microorganisms release and exchange metabolites, promoting optimal development and impacting the growth and acidification of fermentation systems [3]. For example, in a protocooperative relationship, *L. rhamnosus* expresses the extracellular proteinase PrtR [33], releasing and accumulating peptides in the medium [34] by other *Lactobacillus* species. Furthermore, *L. plantarum* and *P. acidilactici* possess a biosynthetic pathway for lysine, which *L. brevis* lacks. Lysine is crucial as a cell wall constituent and an enzymatic substrate in metabolic pathways [35,36,37]. The combination of LAB in a fermentation medium could be pivotal for the development and viability of probiotics in producing symbiotic fermented foods.

During fermentation, LAB utilizes sugars (hexoses and pentoses) to produce lactic acid, decreasing pH [38,39,40]. Adjustments such as increasing pH are also necessary to enhance bacterial growth during fermentation [41]. Li et al. (2021) [42] previously reported a significant increase in LAB counts, exceeding 10 log CFU/mL, in blackberry juices using four *L. plantarum* strains and five *L. fermentum* strains, with a 35% increase in lactic acid production. In our study, a more significant reduction in pH was observed in the juice without whey and LAB consortium 1 (BJ/C1) at 24 and 48 h of fermentation, with an average reduction of up to 46%. This is associated with increased lactic acid at 24 h (75%) and 48 h (83%). However, the pH was lower in juice samples containing whey (BJWH) despite higher lactic acid production in BJWH/C1 and BJWH/C2 compared to the BJ and WH samples (Table 1). A similar relationship between pH changes and increased lactic acid production has been reported in whey fermentation with *L. acidophilus*, *L. delbrueckii* subsp. *bulgaricus* and *S. thermophilus* [30], wolfberry, and longan juice with *L. paracasei* and *L. lactis* subsp. *lactis* [42].

The BJWH/C1 and C2 samples showed a significant increase (*p* < 0.05) in bacterial count during fermentation, with exponential and continuous growth observed for up to 16 h (Figure 2A). This was accompanied by higher lactic acid production and a notable decrease in pH (Figure 3A). Similar results were reported for whey fermented with *L. casei* and *L. rhamnosus*, where exponential biomass growth occurred in the second fermentation stage, likely due to the consumption of lactose and galactose during the first 15 h of exponential growth [43]. Additionally, Wang et al. [44] noted that *L. acidophilus* JYLA-16 (La), *L. plantarum* JYLP-375 (Lp), and *L. rhamnosus* JYLR-005 (Lr) preferentially utilize glucose over fructose as a carbon source during fermentation. Therefore, adding milk whey protein to the fruit juice provides an additional substrate for fermentation, enhancing LAB growth. This effect was similarly observed in pomegranate juice supplemented with whey and fermented with *L. plantarum*, leading to increased LAB growth [45].

The superior performance of the BJWH/C2 system in kinetic parameters can be attributed to a combination of environmental factors and the complex interactions between microorganisms, particularly through protocooperation and synergism. Olvera-Rosales et al. (2023) [3] demonstrated that a co-culture system involving *Lactobacillus rhamnosus* GG and *Streptococcus thermophilus* in whey surpasses monocultures in both specific growth rate (µ) and generation time (g). This enhanced efficiency is reflected in higher lactic acid production and elevated levels of free amino groups [28]. Additionally, co-culture systems in milk fermentation are well documented to significantly enhance the production yields of lactic acid and secondary metabolites like peptides [34]. These findings highlight the crucial role of microbial interactions in optimizing fermentation processes and improving the overall yield of desired compounds.

During storage at 4 °C for 28 days, a significant decrease in LAB counts was observed during the first week, likely due to the adaptation of LAB in the BJWH/C1 and BJWH/C2. Notably, the LAB count remained above 9 Log CFU/mL by the end of the storage period, suggesting potential therapeutic benefits. Previous studies have shown that LAB mixtures in food matrices can exhibit heterogeneous growth behavior during storage at 4 °C [9,46]. However, by the end of 28 days, the strains survived at desirable and therapeutic levels (6–8 Log CFU/mL), depending on their specific adaptation conditions [46,47,48]. In consortia of LAB strains *L. acidophilus*, *L. casei*, and *L. plantarum*, added to ferment apple juice without additional nutrients, the maximum growth during fermentation reached 7.5 to 8.3 Log CFU/mL. However, during storage for 20 to 30 days, viable cell counts of *L. casei* rapidly decreased to 5.4 ± 0.04 Log CFU/mL, followed by *L. acidophilus* (5.6 ± 0.13 Log CFU/mL) and *L. plantarum* (5.7 ± 0.13 Log CFU/mL) [49].

During storage at 4 °C, lactic acid and physicochemical parameters (viscosity, color, soluble solids, reducing sugars) remained stable, with only slight variation observed in antioxidant capacity, total phenolic, and anthocyanin levels. The use of multiple LAB strains can enhance lactic acid production due to their homofermentative and heterofermentative characteristics, as well as the activation of various metabolic pathways (EMP, pentose phosphate, and phosphoketolase), depending on the strains used [50]. The increase in lactic acid production was particularly pronounced in BJWH/C1 and BJWH/C2.

Additionally, the total solids in a fermented product are influenced by differences in the activated enzyme systems and the microorganisms’ ability to utilize matrix substrates [9]. As LAB consumes sugars, producing lactic acid and increased biomass can help maintain a balance in total solids [51]. Conversely, the reduction in soluble solids was more significant in the WH samples compared to BJ and BJWH at 24 and 48 h, as using protein substrates produces fewer soluble soils. The changes observed in BJ and BJWH are associated with a significant increase in lactic acid, over 45%, and a reduction of up to 48.6% in reducing sugars and bacterial growth.

During storage, the trends observed in lactic acid and pH remained consistent, while the content of reducing sugars decreased, and soluble solids stayed the same. This pattern mirrored the changes observed during fermentation. A similar decrease in pH during storage was reported for carrot juice supplemented with whey and fermented with kefir grains over 21 days [52]. In passion fruit juice fermented with *Lactobacillus plantarum*, the reduction in pH was attributed to the combined effects of the strains’ post-acidification capacities and the buffering power of the substrate [53]. Additionally, LAB at low temperatures (4 °C) continue a slow fermentation process to meet their metabolic needs, resulting in ongoing production and consumption of metabolites during this period [54,55].

Lactic acid and pH levels correlated with fermentation or storage time, with correlation coefficients exceeding 0.97. This indicates a highly significant relationship between these parameters and time. The literature suggests that R^2^ values close to 1 demonstrate a robust positive relationship between the variables, as seen in kimchi fermentation studies [56]. This strong correlation enables effective monitoring of the fermentation and storage process. Specifically, as the fermentation or storage time advances, lactic acid levels typically rise while pH decreases. These changes reflect the usual dynamics of fermentation, where increased lactic acid production lowers the medium’s pH. This predictive relationship serves as a valuable tool for tracking process progress and can also be utilized to optimize and control the quality of the final product over time.

During fermentation, the physical stability of BJWH/C1 and BJWH/C2 improved, while viscosity decreased significantly by 57% for BJWH/C1 and 53% for BJWH/C2. Both properties remained stable throughout storage. The food matrix’s structure influences the interplay between stability and viscosity. For instance, a thick and porous gel was observed in the fermentation of milk with strawberry juice using an initial kefir inoculum. However, a protein network with smaller pores enhanced stability and increased viscosity [57]. On the other hand, in a mixed juice (beet, carrot, apple, and strawberry) fermented with kefir grains, the viscosity initially increased within the first 12 h. Still, it later decreased due to the hydrolysis of exopolysaccharides by glycohydrolase [58]. This indicates that LAB-fermented products’ physical stability and viscosity are largely determined by the strains’ ability to integrate into the food matrix and their metabolic activities.

In the color analysis of BJWH/C1 and BJWH/C2 juices during fermentation, a shift from violet to red tones was observed at 16 h. During storage, the color parameters remained stable. This color change is linked to the stability of anthocyanins under varying pH levels, as anthocyanin cations remain stable in acidic conditions [20,53]. For BJWH/C2 samples, the color was red when the pH was below 7, violet between pH 7 and 8, and blue when the pH exceeded 11. Color variations in matrices containing antioxidants are also influenced by the concentration of glycosylated polyhydroxyl (violet) or polymethoxy (red) groups. Most anthocyanins have two, three, or more sugars attached as oligosaccharide side chains [53].

Lactic acid fermentation of antioxidant-rich foods affects these compounds, with changes dependent on the specific strain and the matrix (fiber, protein) in which they are present. During fermentation, a decrease in phenolic compounds was observed in all samples. This reduction can be attributed to thermal processing, which induces physical and chemical reactions, including the breakdown of bound phenolic compounds and their transformation into other forms [59]. LAB are known to metabolize phenolic compounds [60], possibly due to the biotransformation of these compounds through LAB reductases, decarboxylases, and glycosidases [49,61]. Additionally, LAB-produced hydrolases may hydrolyze phenolic complexes into simpler forms and disrupt cell walls, releasing antioxidant compounds, as seen in the co-fermentation of longan and goji juice [42]. In storage, however, sample BJWH/C2 (*L. casei* + *L. ramnhosus*) showed an increased total phenolic content. Phenolic acid decarboxylases can catalyze the non-oxidative decarboxylation of phenolic acids to generate P-vinyl derivatives [62].

The fermentation of a drink made from blackberry juice and whey demonstrated that phenolic compounds and anthocyanins became more stable after fermentation with *L. plantarum* and *L. casei*. Binding anthocyanins or phenolic hydroxyl groups stabilized NH, CN, and C [20]. In apple juice fermented with *L. acidophilus*, *L. casei*, and *L. plantarum*, the total phenolic content decreased after 72 h of fermentation and during the first 10 days of storage [49], with stability observed thereafter until day 20. Similarly, Wang et al. [63] reported on the fermentation of mango juice with *L. bulgaricus*, *S. thermophilus* 6063, and *L. plantarum*.

During fermentation, the anthocyanin content remained stable in the LAB-inoculated samples (BJWH/C1 and BJWH/C2). There was also a slight decrease in BJWH/C1 during storage and a more pronounced decrease in BJWH/C2 starting from day 21. In contrast, blackberry juice without an inoculum significantly reduced anthocyanins from day 14. In yogurt supplemented with 5% freeze-dried mulberry fruit juice (FDMJ), the anthocyanin content increased over 35 days (from 36.49 to 40.65 mg CGE cyanidin-3-glucoside/100 g) [64]. However, antioxidant activities decreased, likely due to the degradation of phenolic compounds or increased milk protein–polyphenol interactions [65]. LAB-produced-glucosidases contribute to reductions in anthocyanins during fermentation by breaking glycosidic bonds [27].

The radical scavenging capacity was measured using ABTS and DPPH assays to assess antioxidant activity. The BJWH/C1 and BJWH/C2 samples retained ABTS activity during the first eight hours of fermentation, followed by a decrease after 12 h. In contrast, DPPH assays showed increased antioxidant capacity during fermentation, especially in BJWH/C1, with activity maintained for the first 14 days of storage (Figure 6D). These antioxidant properties are attributed to proton donation and the samples’ acidic and phenolic hydroxyl groups. Similar findings have been reported for fermented chokeberry [5] and mulberry pomace [66], which experienced a decrease in phenolic and anthocyanin contents during fermentation but showed increased ABTS and DPPH scavenging on the first day of fermentation.

One limitation of this study is the lack of analysis of the profiles of sugars and amino acids at the beginning and end of fermentation. Additionally, there is limited research on the effects of co-cultures or consortia, with most studies focusing on individual LAB and *Saccharomyces* strains. Future research should also explore how adding isolated whey to the medium might affect sensory preferences. Despite this, our study found that including whey enhances LAB growth conditions, resulting in good stability and antioxidant capacity during storage at low temperatures.

## 4. Materials and Methods

### 4.1. Materials

The blackberries were sourced from Atotonilco, Hidalgo, Mexico, and the milk whey (100% isolated whey protein) was purchased from HolixLab, Hilmar ingredients® (Guadalajara, Jalisco, Mexico). The autochthonous strains used (*Lactiplantibacillus plantarum* and *Pediococcus acidilactici* from aguamiel, *Levilactobacillus brevis* from pulque and *Lacticaseibacillus rhamnosus* and *Lacticaseibacillus casei* from fermented milk [23]) were isolated and provided by the Academic Area of Nutrition of Autonomous University of Hidalgo State, México.

Rogosa and Sharpe (MRS) agar and broth from MERCK (Rahway, NJ, USA) and Difco (Sparks, MD, USA), respectively, were utilized for culturing. The reagents used for principal assays included sodium hydroxide (NaOH Labessa, Ciudad De León, Guanajuato, Mexico), Folin–Ciocalteu reagent (Sigma-Aldrich, Louis, MO, USA), sodium carbonate (Meyer, Vallejo, CA, USA), gallic acid (Chem-Cruz, Dallas, TX, USA), potassium chloride (Meyer, Vallejo, CA, USA), sodium acetate (Meyer, Vallejo, CA, USA), hydrochloric acid (Meyer, Vallejo, CA, USA), ammonium 2,2′-azino-bis-(3-ethylbenzothiazoline-6-sulfonate (ABTS, Sigma-Aldrich, Louis, MO, USA), potassium persulfate (Sigma-Aldrich, Louis, MO, USA), ascorbic acid (Meyer, Vallejo, CA, USA), 2,2-Diphenyl-1-Picrylhydrazyl (DPPH, Sigma-Aldrich, Louis, MO, USA), absolute ethyl alcohol (Meyer, Vallejo, CA, USA), 6-hydroxy-2,5,7,8-tetramethylchroman-2-carboxylic acid (Trolox, Sigma-Aldrich, Louis, MO, USA).

### 4.2. Blackberry Juice and Whey Preparation

Blackberry fruit (*Rubus fruticosus*) was sourced locally from Atotonilco El Grande, Hidalgo, Mexico. The method outlined by Cervantes-Elizarrarás [67] was followed to ontain clarified juice. The fruits were washed, disinfected, ground, and filtered with a conventional strainer. The juice was then clarified through centrifugation (10,000 rpm for 30 min at 4 °C) and treated with thermoultrasound under optimal conditions (50 ± 1 °C, 17 ± 1 min at 80% amplituded). After processing, the juice was frozen at −35 °C until needed. The whey was prepared by dissolving at 9.3 g/L of water and sterilized before use (121 °C for 15 min).

### 4.3. Juice Fermentation

Blackberry juice (BJ) and whey (WH) were mixed in a 1:1 ratio to produce BJWH solution, and the pH of all samples (BJ, WH, and BJWH) was adjusted to 6 using 1 M NaOH. The samples were inoculated with LAB strains previously activated on MRS agar (24 h at 37 °C) and growth in 5 mL of MRS broth under the same conditions. The biomass was collected by centrifugation (10,000 rpm for 15 min), resuspended in the fermentation matrices, and adjusted to an approximate initial count of 9 Log CFU/mL. Inoculation was performed with the following consortia: consortium 1 (C1) containing *Lactiplantibacillus plantarum*, *Pediococcus acidilactici* and *Levilactobacillus brevis*; consortium 2 (C2) containing of *Lacticaseibacillus rhamnosus* and *Lacticaseibacillus casei*; and negative control (C-) without LAB. The samples were incubated at 37 °C for 48 h, with 20 mL aliquots taken 0, 24, and 48 h during the initial phase. Testing continued for 32 h in the subsequent phase, with aliquots collected every 4 h. For storage analysis, the fermented samples were kept in the dark at 4 °C, with aliquots taken on days 3, 7, 14, 21, and 28. All aliquots were centrifuged (10,000 rpm for 15 min at 4 °C) and the supernatants were stored at −32 °C to analyze reducing sugars and antioxidant properties.

### 4.4. Microbiological Analysis of Fermented Juice

Microbiological analysis was conducted using the micro drop method described by Strahsburger, Retamales, Estrada, and Seeger [68]. The fermented juice samples’ serial dilutions (1:10) were prepared in a peptone water solution for microbial counting. Aliquots were then inoculated onto MRS agar and incubated at 37 °C for 36 h. The results were logarithms of ten colony-forming units per milliliter (Log10 CFU/mL).

### 4.5. Kinetic Parameters at the Time of Fermentation

The kinetic parameters were determined using Monod’s mathematical model [69], which describes the growth of microorganisms during the exponential phase. The initial (N_0_) and final (N_x_) concentrations of growth were measured during the logarithmic phase, corresponding to the initial time (t_0_) and final time (t_x_). The increases in cell density (A), growth rate (μ), generation time (g) and growth constant (k) were calculated using the following equations:

A = LogN_x_ − LogN_0_

μ = [In(N_x_) − In(N_0_)]/(t_x_ − t_0_)

g = In(2)/μ

k = 1/g

### 4.6. pH, Titratable Acidity and Soluble Solids of Fermented Juice

The pH was measured using a potentiometric method following standard procedures. Titratable acidity was determined by titrating a 1:10 dilution of the juice with deionized water using 0.01 N NaOH until a pH of 8.2 was reached. The titratable acidity was expressed as a percentage equivalent to lactic acid. Total soluble solids (°Brix) were measured with a manual ATC refractometer by placing a drop of the juice on the prism, calibrating, and then taking the reading. Non-linear regression analysis was performed on the lactic acid and pH variables for fermentation (48 h) and storage (28 days), using GeoGebra Classic (version 6.0.851.0, https://www.geogebra.org/classic?lang=es, 12 July 2024) to model polynomial behavior.

### 4.7. Stability and Viscosity of Fermented Juice

The stability percentage was determined using the following procedure: 10 g of sample (sW) was weighed and centrifuged at 3500 rpm (Hamilton, model V65000, Montvale, NJ, USA) for 30 min. The precipitate (SW) was decanted and weighed. Stability was calculated using the equation below and expressed as a percentage.

Stability (%) = 100 − [(SW/sW) × 100]

Viscosity was measured using the method described by Díaz-Jiménez, Sosa-Morales, and Vélez-Ruiz [70] with a Brookfield DV3T viscometer (Manassas, VA, USA), employing needle LV-4 at 60 rpm. The readings were taken in centipoises (cP). A 30 mL aliquot was used, and measurements were started after 30 s, at which point the cP values stabilized.

### 4.8. Measure of the Color of Fermented Samples

Color measurements were taken using a colorimeter (Minolta CM-80, 500 SM-508D, Osaka, Japan Co., Ltd.) with a D65 illuminant and a 10° observation angle. Then, 5 mL of fermented juice was placed in a container for analysis. The color parameters were recorded using the CIE—L* a* b* values, where L* indicates lightness (L = 0 for black and L = 100 for white), a* measures the chromaticity from green (−) to red (+), and b* measures the chromaticity from blue (−) to yellow (+). The colorimeter also provided the hue angle (h) and chroma (C) [71].

### 4.9. Antioxidant Properties

#### 4.9.1. Total Phenolic Content

Total phenolic content (TPC) was measured following the method described by Stintzing et al. [72]. In brief, 100 µL of the sample was mixed with 500 µL of 1:10 diluted Folin–Ciocalteu reagent. After adding 400 µL of 7.5% sodium carbonate, the mixture was incubated at room temperature for 30 min. The absorbance was then measured at 765 nm using a microplate lector (Power Wave XS UV-Biotek Gen 5 software (version 3.16.10), KC Junior, Winooski, VT, USA). Gallic acid served as the reference standard, and the results were expressed as milligrams of gallic acid equivalents per 100 milliliters (mg GAE/100 mL).

#### 4.9.2. Anthocyanins

The anthocyanin content was measured using the method outlined by Wallace and Giusti [73]. Two buffers were prepared: potassium chloride at 0.025 M and pH 1 (0.466 g in 250 mL of distilled water) and sodium acetate at 0.4 M and pH 4.5 (8.203 g in 250 mL of distilled water), with pH adjusted using 6 M HCl. A 4.5 mL aliquot of either buffer was mixed with 0.5 mL of the sample, and the mixture was allowed to stand for 15 min at room temperature and in complete darkness. Absorbance was then measured using a microplate lector (Power Wave XS UV-Biotek Gen 5 software (version 3.16.10) KC Junior, Winooski, VT, USA) at 510 and 700 nm wavelengths with the respective buffer. Results were expressed as milligrams of Cyanidin-3-glucoside per 100 milliliters of the sample (mg Cy-3-Gl/100 mL).

#### 4.9.3. Antioxidant Activity by ABTS

Antiradical capacity was assessed following the method described by Kuskoski, Asuero, Troncoso, Mancini-Filho, and Fett [74]. An ABTS stock solution was prepared using 7 mmol/L ABTS and 2.45 mmol/L potassium persulfate and stored at a dark room temperature for 16 h. This stock solution was diluted with deionized water until an absorbance of 0.70 ± 0.10 at 754 nm was achieved. A 20 µL aliquot of the sample was added to 980 µL of the diluted ABTS solution, and absorbance readings were taken after 7 min incubation at room temperature. The absorbance was measured at 754 nm using a microplate reader (Power Wave XS UV-Biotek Gen 5 software (version 3.16.10), KC Junior, Winooski, VT, USA). Antioxidant capacity was expressed as milligrams of vitamin C equivalent antioxidant capacity per 100 milliliters (mg VCEAC/100 mL) of the fermented juice.

#### 4.9.4. Antioxidant Activity by DPPH

Antiradical activity was assessed using DPPH radicals [75]. A stable DPPH radical solution was prepared in ethanol (7.4 mg/100 mL). In Eppendorf tubes, 100 µL of the extract was mixed with 500 µL of the DPPH solution and allowed to react for one hour at room temperature. Absorbance was then measured at 520 nm using a microplate reader (Power Wave XS UV-Biotek Gen 5 software (version 3.16.10), KC Junior, Winooski, VT, USA). The results are expressed as the fermented juice’s micromoles of Trolox equivalents per 100 milliliters (µmol TE/100 mL).

### 4.10. Statistical Analysis

All results are presented as the mean and standard deviation (mean ± SD) of three independent experiments (n = 3) with a significance level of *p* < 0.05. Comparisons were made for the same strain or consortium at different fermentation times (0, 24, and 48 h) and storage periods (3, 7, 14, 21, and 28 days). Additionally, analyses were conducted between different strains or consortia across fermentation and storage time. Data were analyzed using one-way analysis of variance (ANOVA), with two-way ANOVA applied for bacterial growth during fermentation (48 h) and storage (28 days). Kinetic parameters were compared using Student’s *t*-test and post hoc Tukey test, with statistical analyses performed using SPSS v. 19 software.

## 5. Conclusions

This study underscores the significant advantages of incorporating whey protein into fruit-based beverages, particularly highlighting its impact on enhancing the antioxidant content and maintaining probiotic viability during refrigerated storage. This approach provides a sustainable alternative for using blackberries and showcases the synergistic effects between probiotics, whey-derived peptides, and phenolic compounds, which act as potential prebiotics.

Statistical analysis reveals that fermenting blackberry juice with added whey protein and two or three strains of lactic acid bacteria (LAB) results in continuous and linear growth patterns, surpassing the fermentation outcomes of blackberry juice or whey alone. The regression analysis further confirms a strong correlation between pH and lactic acid production, dependent on the time, throughout fermentation and subsequent refrigerated storage. These findings indicate that the effectiveness of these fermentations depends on the specific proteolytic systems of the bacteria involved and their ability to adapt to the medium.

Moreover, the lactic acid fermentation process contributes to the stability of the beverage’s physicochemical properties, notably enhancing its viscosity and color characteristics in both consortia (BJWH/C1 and BJWH/C2). Additionally, the fermentation of BJWH/C1 and BJWH/C2 during storage successfully preserves anthocyanins, ensuring sustained antioxidant potential over 28 days at low temperatures.

These advancements suggest that such formulations could improve consumer acceptability and offer potential health benefits. Future research is recommended to explore these potential benefits further and optimize the formulation for enhanced performance.

## Figures and Tables

**Table 1:** Physicochemical properties after fermentation in the two consortia of LAB were inoculated in the experimental samples (WH, BJ, and its combination).

Sample	pH			% Lactic Acid			Total Soluble Solids (°Brix)			Reducing Sugars (g/100 mL)		
	0 h	24 h	48 h	0 h	24 h	48 h	0 h	24 h	48 h	0 h	24 h	48 h
WH/												
C1	5.7 ± 0.00 ^D,a^	4.1 ± 0.00 ^BC,b^	4.0 ± 0.01 ^E,c^	0.02 ± 0.00 ^F,b^	0.07 ± 0.01 ^G,a^	0.07 ± 0.00 ^FG,a^	0.9 ± 0.06 ^D,a^	0.6 ± 0 ^F,b^	0.4 ± 0.00 ^G,c^	Nd	Nd	Nd
C2	5.9 ± 0.01 ^BC,a^	3.8 ± 0.03 ^C,b^	3.8 ± 0.04 ^F,b^	0.02 ± 0.00 ^F,b^	0.05 ± 0.00 ^GH,a^	0.06 ± 0.00 ^G,a^	0.8 ± 0.06 ^D,a^	0.4 ± 0 ^G,b^	0.0 ± 0.00 ^H,c^	Nd	Nd	Nd
C-	5.7 ± 0.01 ^D,a^	5.7 ± 0.00 ^A,b^	5.7 ± 0.03 ^B,b^	0.02 ± 0.00 ^F,b^	0.03 ± 0.00 ^H,a^	0.03 ± 0.00 ^G,ab^	1.0 ± 0.00 ^D^	1.0 ±0.00 ^E^	1.0 ±0.00 ^F^	Nd	Nd	Nd
BJ/												
C1	5.9 ± 0.02 ^C,a^	3.6 ± 0.20 ^C,b^	3.6 ± 0.04 ^H,b^	0.4 ± 0.00 ^A,c^	1.5 ± 0.02 ^A,a^	2.3 ± 0.02 ^A,b^	10 ± 0.06 ^A,a^	9.07 ± 0.1 ^B,b^	8.9 ± 0.06 ^C,b^	9.5 ± 0.00 ^A,a^	7.9 ± 0.10 ^B,c^	8.4 ± 0.08 ^B,b^
C2	6.1 ± 0.04 ^A,a^	4.7 ± 0.02 ^B,b^	4.05 ± 0.00 ^D,c^	0.3 ± 0.00 ^B,c^	0.7 ± 0.01 ^D,b^	1.4 ± 0.01 ^B,a^	9.6 ± 0.30 ^AB,a^	9.9 ± 0.1 ^A,a^	9.5 ± 0.00 ^B,a^	8.3 ± 0.08 ^C,a^	6.1 ± 0.10 ^C,c^	6.5 ± 0.03 ^C,b^
C-	5.9 ± 0.01 ^B,a^	5.8 ± 0.03 ^A,b^	5.6 ± 0.00 ^C,c^	0.3 ± 0.01 ^B,b^	0.3 ± 0.00 ^E,b^	0.4 ± 0.01 ^E,a^	9.3 ± 0.20 ^B,b^	10 ± 0.06 ^A,a^	10 ± 0.00 ^A,a^	8.9 ± 0.07 ^B,a^	8.6 ± 0.10 ^A,b^	9.03 ± 0.17 ^A,a^
BJWH/												
C1	5.4 ± 0.00 ^F,a^	3.7 ± 0.01 ^C,b^	3.5 ± 0.01 ^I,c^	0.2 ± 0.00 ^C,c^	0.9 ± 0.05 ^B,b^	1.2 ± 0.01 ^C,a^	6.0 ± 0.00 ^C^	5.0 ± 0.00^D^	5.0 ± 0.00 ^E^	3.5 ± 0.01 ^E,a^	2.6 ± 0.00 ^D,b^	1.8 ± 0.00 ^F,c^
C2	5.6 ± 0.01 ^E,a^	3.8 ± 0.01 ^C,b^	3.7 ± 0.00 ^G,c^	0.2 ± 0.00 ^D,c^	0.8 ± 0.01 ^C,b^	0.9 ± 0.05 ^D,a^	6.0 ± 0.00 ^C^	5.0 ± 0.00 ^D^	5.0 ± 0.00 ^E^	3.5 ± 0.02 ^E,a^	2.5 ± 0.03 ^D,c^	2.7 ± 0.00 ^E,b^
C-	5.6 ± 0.01 ^E,a^	5.8 ± 0.60 ^A,a^	5.8 ± 0.00 ^A,a^	0.1 ± 0.00 ^E,a^	0.1 ± 0.00 ^F,a^	0.13 ± 0.01 ^F,a^	6.0 ± 0.00 ^C^	6.0 ± 0.00 ^C^	6.0 ± 0.00 ^D^	3.6 ± 0.03 ^D,a^	2.6 ± 0.03 ^D.c^	3.4 ± 0.04 ^D,b^

BJ: blackberry juice; BJWH: blackberry juice + whey protein; WH: whey protein; C1: Consortium 1 of *Levilactobacillis brevis*, *Lactiplantibacillus plantarum*, and *Pediococcus acidilactici*, C2: Consortium 2 of *Lacticaseibacillus casei* and *Lacticaseibacillus rhamnosus*; C-: Consortium without LAB inoculum; Nd: not detectable. Values represent the mean ± standard deviation of three experiments (n = 3). Different capital letters indicate a significant difference (*p* < 0.05) between the different strains or consortium for each fermentation time. Different lowercase letters indicate a significant difference (*p* < 0.05) of each strain or consortium between their different fermentation times.

**Table 2:** Kinetic parameters at the time of fermentation (16 h) of blackberry juice supplemented with whey.

Fermentation System	Parameter Value			
	A (Log 10 CFU/mL)	µ (h^−1^)	g	k (g/h)
BJWH/C1	5.39 ± 0.13	0.33 ± 0.00	2.05 ± 0.05	0.48 ± 0.01
BJWH/C2	2.67 ± 0.36 *	0.16 ± 0.02 *	4.20 ± 0.60 *	0.24 ± 0.03 *

Blackberry juice + whey protein inoculated with consortium 1 (*Levilactobacillus brevis*, *Lactiplantibacillus plantarum*, and *Pediococcus acidilactici*, BJWH/C1), consortium 2 (*Lacticaseibacillus casei* and *Lacticaseibacillus rhamnosus*, BJWH/C2) and the negative control without LAB inoculum (BJWH/C-). A: increase in cell density; μ: Grow rate; g: Generation time; k: Growth rate constant. Values represent three experiments’ mean ± standard deviation (n = 3). * Indicates significant differences between the samples.

**Table 3:** Total soluble solids and reducing sugars at the time of fermentation (0–16 h) and storage (3–28 days) of blackberry juice supplemented with whey.

Time	Total Soluble Solids (°Brix) ^1^			Reducing Sugars (g/100 mL)		
	BJWH/C1	BJWH/C2	BJWH/C-	BJWH/C1	BJWH/C2	BJWH/C-
0 h	6	6	6	3.4 ± 0.20 ^AB,a^	2.9 ± 0.10 ^B,a^	3.5 ± 0.20 ^A,bcd^
4 h	6	6	6	2.1 ± 0.20 ^B,d^	2.6 ± 0.10 ^C,ab^	3.7 ± 0.40 ^A,bcd^
8 h	5	5	6	2.6 ± 0.04 ^B,b^	2.1 ± 0.30 ^B,c^	3.3 ± 0.20 ^A,cd^
12 h	5	5	6	2.2 ± 0.01 ^B,cd^	2.2 ± 0.03 ^B,c^	3.2 ± 0.02 ^A,d^
16 h	5	5	6	2.1 ± 0.06 ^B,d^	2.1 ± 0.08 ^B,c^	3.4 ± 0.10 ^A,bcd^
Day 3	5	5	6	2.5 ± 0.10 ^B,bc^	2.0 ± 0.10 ^C,c^	3.7 ± 0.07 ^A,abd^
Day 7	5	5	6	2.2 ± 0.60 ^B,bcd^	2.2 ± 0.04 ^B,c^	3.4 ± 0.01 ^A,bcd^
Day 14	5	5	6	2.1 ± 0.01 ^C,d^	2.45 ± 0.06 ^B,bc^	3.4 ± 0.06 ^A,bcd^
Day 21	5	5	6	2.4 ± 0.08 ^B,bcd^	2.1 ± 0.08 ^C,c^	3.9 ± 0.05 ^A,a^
Day 28	5	5	6	2.5 ± 0.10 ^B,bc^	2.2 ± 0.03 ^B,bc^	3.7 ± 0.10 ^A,ab^

Blackberry juice + whey protein inoculated with consortium 1 (*Levilactobacillus brevis*, *Lactiplantibacillus plantarum*, and *Pediococcus acidilactici*, BJWH/C1), consortium 2 (*Lacticaseibacillus casei* and *Lacticaseibacillus rhamnosus*, BJWH/C2) and the negative control without LAB inoculum (BJWH/C-). Values represent three experiments’ mean ± standard deviation (n = 3); ^1^ In these experiments, the SD was zero. Different capital letters indicate significant differences (*p* < 0.05) between the various strains or consortium for each fermentation time (0–48 h and 3–28 days). Different lowercase letters indicate a significant difference (*p* < 0.05) of each strain or consortium between their different fermentation and storage times.

**Table 4:** Stability and viscosity at the time of fermentation (0–16 h) and storage (3–28 days) of blackberry juice with whey.

Time	Stability (%)			Viscosity (cP)		
	BJWH/C1	BJWH/C2	BJWH/C-	BJWH/C1	BJWH/C2	BJWH/C-
0 h	73.7 ± 0.6 ^B,b^	76.3 ± 0.5 ^A,c^	71.3 ± 0.5 ^C,ab^	64 ± 0.8 ^A,a^	51.2 ± 0.01 ^B,a^	63.7 ± 0.9 ^A,ab^
16 h	84.6 ± 0.5 ^B,a^	87.7 ± 0.5 ^A,a^	73 ± 1 ^C,a^	27.5 ± 1.7 ^B,b^	24.9 ± 0.4 ^B,b^	50.4 ± 1.5 ^A,c^
Day 3	85.3 ± 0.5 ^A,a^	86.6 ± 0.5 ^B,ab^	67.4 ± 0.5 ^C,cd^	22.4 ± 2.2 ^B,b^	23.7 ± 2.5 ^B,b^	57.6 ± 1.9 ^A,bc^
Day 7	85.6 ± 0.5 ^A,a^	85 ± 1 ^A,b^	69.6 ± 0.5 ^B,bc^	26.6 ± 5.2 ^B,b^	23.3 ± 3 ^B,b^	59.6 ± 2.6 ^A,ab^
Day 14	85 ± 0.1 ^B,a^	87 ± 0.07 ^A,ab^	67.3 ± 0.5 ^C,d^	29.7 ± 0.8 ^B,b^	24.1 ± 2.1 ^B,b^	62.5 ± 2.9 ^A,ab^
Day 21	84.7 ± 1.1 ^A,a^	86.4 ± 0.5 ^A,ab^	66.5 ± 1.3 ^B,d^	28.2 ± 0.6 ^B,b^	25.1 ± 2.4 ^B,b^	67.5 ± 5.9 ^A,ab^
Day 28	86.3 ± 0.5 ^A,a^	86 ± 1.7 ^A,ab^	69.7 ± 0.6 ^B,b^	27.8 ± 1 ^B,b^	25.9 ± 0.3 ^B,b^	70.5 ± 0.7 ^A,a^

Blackberry juice + whey protein inoculated with consortium 1 of *Levilactobacillus brevis*, *Lactiplantibacillus plantarum*, and *Pediococcus acidilactici* (BJWH/C1), consortium 2 of *Lacticaseibacillus casei* and *Lacticaseibacillus rhamnosus* (BJWH/C2) and the negative control without LAB inoculum (BJWH/C-). Values represent three experiments with mean ± standard deviation (n = 3). Different capital letters indicate significant differences (*p* < 0.05) between the different strains or consortiums for each fermentation and storage time. Different lowercase letters indicate a significant difference (*p* < 0.05) of each strain or consortium between their different fermentation and storage times.

**Table 5:** Colorimetric characteristics of blackberry juice supplemented with whey and two consortia at the time of fermentation (16 h) and time of storage (28 days).

Parameter	Sample	Fermentation		Storage				
		0 h	16 h	Day 3	Day 7	Day 14	Day 21	Day 28
L	BJWH/C1	33.6 ± 0.50 ^B,b^	35.3 ± 0.40 ^A,a^	36.1 ± 0.40 ^A,a^	35.6 ± 0.20 ^A,a^	34.8 ± 1.20 ^A,ab^	35.2 ± 0.30 ^A,ab^	35.7 ± 0.20 ^A,a^
	BJWH/C2	35.4 ± 0.40 ^A,a^	34.9 ± 0.60 ^A,a^	36.3 ± 0.60 ^A,a^	36.0 ± 0.10 ^A,a^	35.6 ± 0.50 ^A,a^	36.0 ± 0.10 ^A,a^	35.4 ± 0.80 ^A,a^
	BJWH/C-	34.5 ± 0.40 ^AB,a^	33.5 ± 0.08 ^B,a^	34.6 ± 0.60 ^B,a^	34.4 ± 0.50 ^B,a^	34.3 ± 0.50 ^A,a^	34.0 ± 0.50 ^B,a^	34.6 ± 10 ^A,a^
a*	BJWH/C1	1.3 ± 0.10 ^B,b^	7.8 ± 0.10 ^A,a^	8.3 ± 0.20 ^B,a^	7.9 ± 0.30 ^B,a^	8.4 ± 0.20 ^B,a^	8.1 ± 0.40 ^B,a^	8.2 ± 0.10 ^B,a^
	BJWH/C2	2.5 ± 0.30 ^A,d^	8.1 ± 0.60 ^A,c^	9.5 ± 0.50 ^A,abc^	9.1 ± 0.10 ^A,bc^	10 ± 0.70 ^A,ab^	10.5 ± 0.10 ^A,a^	9.9 ± 0.03 ^A,ab^
	BJWH/C-	1.5 ± 0.20 ^B,a^	1.3 ± 0.30 ^B,a^	1.7 ± 0.30 ^C,a^	1.2 ± 0.03 ^C,a^	1.4 ± 0.10 ^C,a^	1.1 ± 0.02 ^C,a^	1.2 ± 0.04 ^C,a^
b*	BJWH/C1	−0.8 ± 0.06 ^B,a^	−1.3 ± 0.10 ^B,a^	−1.3 ± 0.10 ^B,a^	−1.2 ± 0.10 ^B,a^	−1.2 ± 0.20 ^C,a^	−1.0 ± 0.07 ^C,a^	−1.0 ± 0.06 ^C,a^
	BJWH/C2	−1.4 ± 0.02 ^C,c^	−0.8 ± 0.10 ^B,bc^	−1.0 ± 0.10 ^B,bc^	−0.7 ± 0.10 ^AB,ab^	−0.5 ± 0.03 ^B,ab^	−0.4 ± 0.10 ^B,a^	−0.5 ± 0.07 ^B,ab^
	BJWH/C-	−0.6 ± 0.06 ^A,c^	−0.1 ± 0.01 ^A,bc^	−0.1 ± 0.30 ^A,abc^	−0.1 ± 0.01 ^A,abc^	0.04 ± 0.20 ^A,abc^	0.3 ± 0.06 ^A,ab^	0.3 ± 0.06 ^A,a^
Chroma	BJWH/C1	1.7 ± 0.20 ^B,b^	7.9 ± 0.10 ^B,a^	8.4 ± 0.30 ^B,a^	7.9 ± 0.30 ^B,a^	8.4 ± 0.1 ^B,a^	8.2 ± 0.40 ^B,a^	8.2 ± 0.10 ^B,a^
	BJWH/C2	2.8 ± 0.20 ^A,d^	8.2 ± 0.60 ^A,c^	9.6 ± 0.50 ^A,abc^	9.1 ± 0.10 ^A,bc^	10 ± 0.7 ^A,ab^	10.5 ± 0.10 ^A,a^	10 ± 0.03 ^A,abc^
	BJWH/C-	1.7 ± 0.10 ^B,a^	1.4 ± 0.30 ^B,a^	1.7 ± 0.30 ^C,a^	1.2 ± 0.03 ^C,a^	1.4 ± 0.1 ^C,a^	1.1 ± 0.01 ^C,a^	1.2 ± 0.05 ^C,a^
Hue	BJWH/C1	−27.4 ± 2.20 ^A,b^	−8.5 ± 0.70 ^A,a^	−9.2 ± 0.70 ^A,a^	−8.5 ± 0.40 ^A,a^	−7.5 ± 1.40 ^A,a^	−7.3 ± 0.10 ^B,a^	−6.7 ± 0.30 ^C,a^
	BJWH/C2	−26.3 ± 1.80 ^A,b^	−6.1 ± 0.60 ^A,a^	−5.7 ± 0.60 ^A,a^	−4.7 ± 0.90 ^A,a^	−2.7 ± 0.20 ^A,a^	−2.2 ± 0.60 ^B,a^	−3.2 ± 0.40 ^B,a^
	BJWH/C-	−20.7 ± 0.30 ^A,c^	−7.4 ± 1.80 ^A,cd^	−3.2 ± 0.20 ^A,abc^	−7.3 ± 0.90 ^A,c^	1.9 ± 0.10 ^A,abc^	15.3 ± 2.60 ^A,ab^	17 ± 1.60 ^A,a^
Sistema color	BJWH/C1	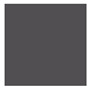	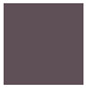	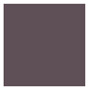	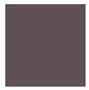	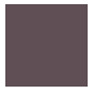	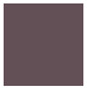	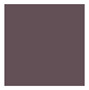
	BJWH/C2	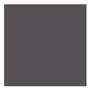	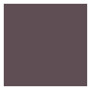	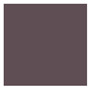	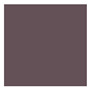	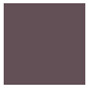	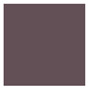	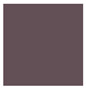
	BJWH/C-	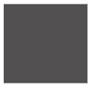	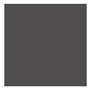	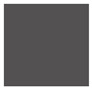	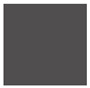	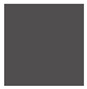	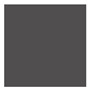	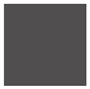

L: luminosity. Blackberry juice + whey inoculated with consortium 1 of *Levilactobacillus brevis*, *Lactiplantibacillus plantarum*, and *Pediococcus acidilactici* (BJWH/C1), consortium 2 of *Lacticaseibacillus casei* and *Lacticaseibacillus rhamnosus* (BJWH/C2), and the negative control without LAB inoculum (BJWH/C-). Values represent three experiments with mean ± standard deviation (n = 3). Different capital letters indicate significant differences (*p* < 0.05) between the different strains or consortiums for each fermentation and storage time. Different lowercase letters indicate a significant difference (*p* < 0.05) between fermentation and storage time of each strain or consortium.

## Data Availability

The data presented in this study are available on request from the corresponding author.
